# Color painting predicts clinical symptoms in chronic schizophrenia patients via deep learning

**DOI:** 10.1186/s12888-021-03452-3

**Published:** 2021-10-22

**Authors:** Hui Shen, Shui-Hua Wang, Yi Zhang, Haixia Wang, Feng Li, Molly V. Lucas, Yu-Dong Zhang, Yan Liu, Ti-Fei Yuan

**Affiliations:** 1 Shanghai Key Laboratory of Psychotic Disorders, Shanghai Mental Health Center, Shanghai Jiao Tong University School of Medicine, Shanghai, China; 2 School of Computing and Mathematical Sciences, University of Leicester, Leicester, LE1 7RH UK; 3 Wu Tsai Neurosciences Institute, Stanford University, Stanford, CA USA; 4 Co-innovation Center of Neuroregeneration, Nantong University, Nantong, Jiangsu China; 5 Translational Research Institute of Brain and Brain-Like Intelligence, Shanghai Fourth People’s Hospital Affiliated to Tongji University School of Medicine, Shanghai, China

**Keywords:** Deep learning, ResNet, Schizophrenia, Color perception, Painting

## Abstract

**Background:**

Individuals with psychiatric disorders perceive the world differently. Previous studies indicated impaired color vision and weakened color discrimination ability in psychotic patients. Examining the paintings from psychotic patients can measure the visual-motor function. However, few studies examined the potential changes in the color painting behavior in these individuals. The current study aims to discriminate schizophrenia patients from healthy controls (HCs) and predict PANSS scores of schizophrenia patients according to their paintings.

**Methods:**

In the present study, we retrospectively analyzed the paintings colored by 281 chronic schizophrenia patients and 35 HCs. The images were scanned and processed using series of computational analyses.

**Results:**

The results showed that schizophrenia patients tend to use less color and exhibit different strokes compared to HCs. Using a deep learning residual neural network (ResNet), we were able to discriminate patients from HCs with over 90% accuracy. Further, we developed a novel convolutional neural network to predict PANSS positive, negative, general psychopathology, and total scores. The Root Mean Square Error (RMSE) of the prediction was low, which indicates higher accuracy of prediction.

**Conclusion:**

In conclusion, the deep learning paradigm showed the large potential to discriminate schizophrenia patients from HCs based on color paintings. Besides, this color painting-based paradigm can effectively predict clinical symptom severity for chronic schizophrenia patients. The color paintings by schizophrenia patients show potential as a tool for clinical diagnosis and prognosis. These findings show potential as a tool for clinical diagnosis and prognosis among schizophrenia patients.

## Background

Individuals with psychiatric disorders accompany different perception abilities [1]. There is strong evidence that psychotic patients showed overall abnormalities in the two constructs of visual perception, gain control and integration [2]. Specifically, previous studies reported that color vision is impaired in patients taking antipsychotic medication, potentially due to altered dopaminergic transmission [3]. On the other hand, psychotic patients exhibited aberrant visual aftereffects [1], differential color priming effect [4], and weakened color discrimination ability [5]. What is more, the self-report altered visual perception can predict which individuals convert to schizophrenia [6]. One approach to measuring visual-motor function is to look at paintings from the clinical subjects. For instance, paintings from Vincent van Gough were considered to have a distinct combination of colors than other artists; interestingly, psychotic patients showed reduced sensitivity to visual feature manipulations in these paintings [7]. Additionally, one study reported that paintings from artists with psychosis were associated with altered spatial frequency content [8].

Deep learning is a new method to identify and transfer artistic styles to painted images, as reported in LA Gatys, AS Ecker and M Bethge [9]. Besides, F Luan, S Paris, E Shechtman and K Bala [10] developed a deep-learning approach to photographic style transfer that handles a large variety of image content while faithfully transferring the reference style. Those detected artistic styles can be used in medical diagnosis, e.g., Parkinson’s disease [11]. ResNet is one type of Deep learning and competes with the other networks by taking the advantage of a “shortcut” connection which makes the training of hundreds or even thousands of layers possible. ResNet has been widely applied for object recognition, image classification, and so on.

The present study aimed to explore the differences in color and stroke information between patients from a group of chronic schizophrenia patients and HCs without psychiatric disorders. After validation there are significant differences between groups, we aimed to use the latest deep learning technique to achieve automatic Schizophrenia detection and PANSS score prediction.

## Methods and materials

### Human subjects

The present study recruited 35 healthy subjects (aged 30–80 years old), and 281 schizophrenia adult patients (aged 20–79 years old). All the participants were recruited in Shanghai Jiao Tong University affiliated Shanghai Mental Health Center (Minhang Campus). The subjects were diagnosed with schizophrenia according to the Diagnostic and Statistical Manual of Mental Disorders 4th edition (DSM-IV) by trained psychiatrists using the SCID (Structured Clinical Interview for DSM IV) and had clinically stabilized disease defined by the absence of antipsychotic treatment modifications within the last 2 months. The subjects have no previous training experiences relate to painting and were not color-blinded. The subjects have dementia, epilepsy, or other neurological co-morbidities were excluded from the study. All the participants volunteered to participate in the study and informed consent was obtained. In case a patient lacked the capacity to consent, their legal guardians were invited to provide consent. The study has been approved by ethics committee of medical research at Shanghai Mental Health Center.

Clinical information, including the Positive and Negative Syndrome Scale (PANSS) and medical information, was collected. PANSS composes of three components: (1) Positive (P), (2) Negative (N), (3) cognitive or General Psychopathology. The positive scale measures symptoms such as hallucinations and delusions. Negative scale measures symptoms such as affective deficits and social function impairment. General Psychopathology measures cognitive deficits such as disorientation and active social avoidance. Each item is scored from one to seven points based on the severity of symptoms (1 represents the absence of symptoms, seven represent extremely severe symptoms). The total points of each scale are 49, 49, and 112 for Positive scale, Negative scale, and General Psychopathology, respectively [12]. All methods were carried out in accordance with relevant guidelines and regulations.

**Fig. 1:**
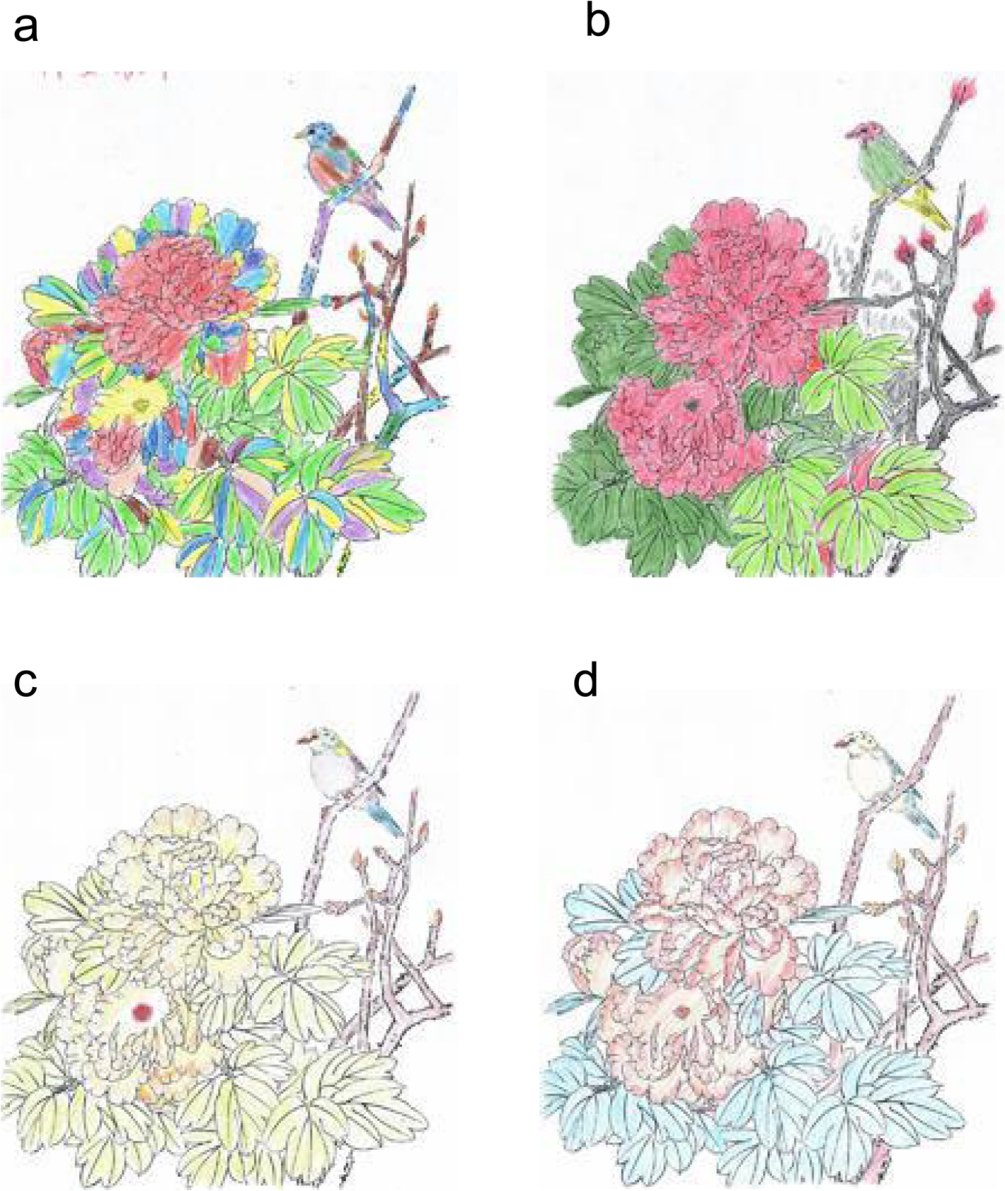
Samples of color painting. a-b are two samples of color painting from healthy subjects. c-d are two samples from schizophrenia patients

For the coloring task, participants were provided a standard template (outlined image) of a bird on a tree with flowers. They were asked to fill in the color using colored pens (available in 12 color choices) with the same instructions. There is no standard answer to this painting test, and the subjects were asked to fill in the color based on personal imagination. Example paintings are shown in Fig. 1.

### Color histogram

The paintings were scanned into images with 300X300 DPI for computer analyses. For each image, we divide the RGB space into 64 colors via a method called "color histogram";, which describes the color distribution in an image and can be built in all color spaces. For digital images, the color distribution is represented by the amounts of pixels of each specific color.

### Accelerator space in Hough transform

We further examined the direction and magnitude of strokes used during the coloring task using the Hough Transform (HT). HT was proposed by [13] as a tool to extract features from images. Features of interest are identified by detecting arbitrary shapes based on their boundary points. The main advantages of HT include the following three points: (1) Each boundary point is processed independently, making HT robust to occlusion or gaps. (2) HT is particularly robust to noise, as noise points cannot continually contribute to any single accumulator’s bins, in which each bin stands for cognate elements in the HT space matrix. (3) This transform can detect more than one instance in a model in a single pass, providing greater computational efficiency.

**Fig. 2:**
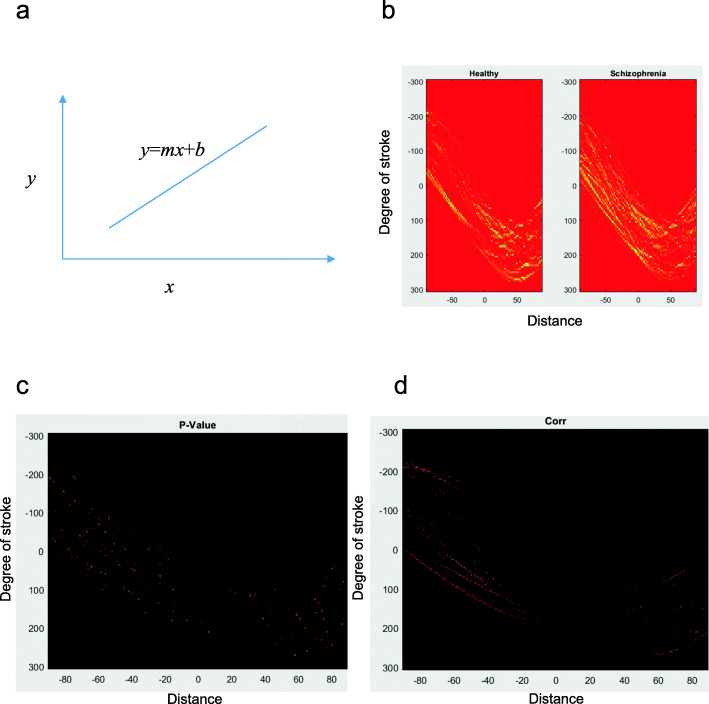
Stroke analysis via Hough Transform. (**a**) Line in a parameter space. For the rest three subimages, X-axis represents the degree of stroke from [−90, 90], the y-axis represents the distance of the line-segment stroke from the origin point. (**b**) Accelerator space is obtained by Hough transform. The color (yellow) means larger values in the accelerator space, while the color (red) means smaller values in the accelerator space. Larger values indicate there are larger possibilities of strokes. (**c**) the results of a two-sample t-test for the strokes between patients and HC (*p*-value), where we set the strictest threshold *p* < 0.001, due to the effect of multiple comparison (FDR correction) (**d**) correlation coefficients, where we only show the correlation coefficient greater than 0.3. The X-axis represents the angle of the strokes. Y-axis represents the distance from the origin to strokes

A general case of HT used for line detection (here, used to detect artists’ strokes): the straight-line *y* = *mx* + *b* is represented by a point (*b*, *m*) in the parameter space, as shown in Fig. 2(a).

However, in the parameter space, the vertical lines proposed the rise to the unbounded values to parameter *m*. Therefore, the Hesse normal form was proposed by RO Duda and PE Hart [14], as shown in (1), to avoid infinity slope.

1 $$ r= xcos\theta + ysin\theta $$

In this equation, *r* stands for the perpendicular distance from the line to the origin, and *θ* represents the angle between the *x*-axis and the line passing through the closest point and origin.

When HT is implemented in the two-dimensional array, called the “accumulator space”, each element is called a bin to detect the existence of the lines. In addition to the length of each line, they are also defined by their polar representation (1).

In this study, we employed the accumulator space to detect the direction and magnitude of strokes used during the coloring task. The basic algorithm of the HT is listed in Table 1. From here, we calculated differences between stroke direction and magnitude between the Schizophrenia patients and HCs.

**Table 1:** Pseudocode of HT

Step 1	Initialize H [*r*, *θ*] = 0.
Step 2	for each edge point *I* [*x*, *y*] in the image for θ = [θ_min_ to θ_max_] * r* = *x* cos*θ + y* sin*θ* H [*r*, *θ*] + =1
Step 3	Find the value of (r*, θ*) where H [*r*, *θ*] is maximum
Step 4	The detected line in the image is given by *r* ∗ = *xcosθ* ∗ + *ysinθ*∗

### Deep learning classification

The above color analysis using the Hough transform identified discernible differences between painting among HCs and Schizophrenia patients. Next, we aimed to identify Schizophrenia patients from HCs in a high-throughput manner, using the colored images alone as input.

Deep learning, also known as deep structured learning, is a technique in which higher-dimensional features can be learned from raw input features. To date, most deep learning models are built using neural networks. In the field of computer vision, neural networks have performed very well in image analysis. Certain networks, such as ResNet, have been trained on exceptionally large datasets, allowing for this high accuracy.

In this study, the size of the data is far from sufficient to train a robust network from scratch. Instead, we used transfer learning, which reapplies knowledge from related tasks to deal with novel problems. We utilized ResNet as the basic model due to its strong performance in ImageNet classification tasks. Additionally, ResNet has been successfully used in overcoming the challenges of vanishing or exploding gradient problems prevalent in very deep networks.

### Transfer learning of ResNet-18

**Fig. 3:**
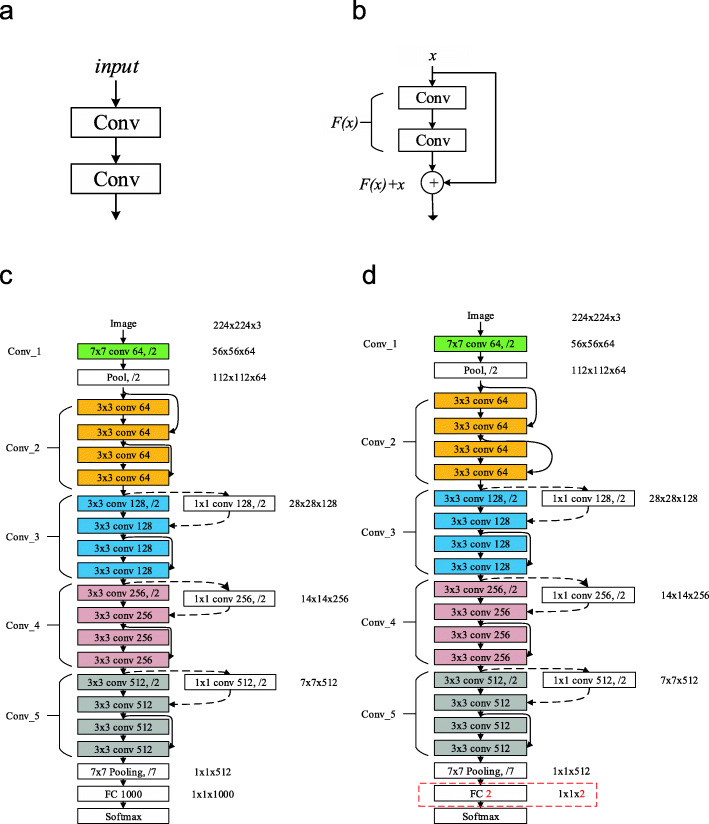
Structural Model of ResNet18. (**a**) Structure of ordinary two conv layers; (**b**) Structure of Residual Block, adding a skip connection from before the first conv layer to the summation operation; (**c**) original structure of ResNet-18; (**d**) Transfer learning of ResNet 18 to predict Schizophrenia from HCs. The red dashed rectangle shows the revision compared to (**c**)

ResNet was proposed in 2015 [15]. With particularly deep CNN architectures, exploding and vanishing gradients become an issue. To deal with this, shortcut connections were used in ResNet. Instead of gradually connecting each layer as shown in Fig. 3(a), ResNet adds skip-connections between neighboring layers, collectively identifying these layers as a “residual block”, as shown in Fig. 3(b). Mathematically, the shortcut connection can be expressed as:

2 $$ y=F(x)+x $$

Because a large dataset is required to train a model of this complexity from the start, we used transfer learning starting with the pretrained ResNet model (ResNet-18) that has been learned how to solve a similar classification problem. The ResNet-18 was pre-trained on the ImageNet project, which classifies 1000 categories of images with more than 14 million samples. Transfer learning has significant advantages, it enables us to train the deep learning model with relatively small data. In this study, we aim to classify the HCs and the schizophrenia patients using binary classification. To accomplish this, we replaced the fully connected layer with 1000 neurons as shown in Fig. 3(c) with a 2-neuron layer as displayed in Fig. 3(d). The structure of all other layers remained the same. The color task images were resized to 224x224x3 to remain consistent with the input-size requirement for ResNet-18.

The unbalanced dataset (35 HCs versus 281 Schizophrenia patients, shown in Table 2) can lead to difficulties in training the network, such as overfitting and poor generalization ability. To solve this problem, the data augmentation method was applied. We divided the dataset into two parts via hold-out validation: The training set contained 20 healthy samples and 266 Schizophrenia samples. We applied 13x oversampling to healthy samples so as to obtained 20 × 13 = 260 healthy samples. The test set consisted of 15 healthy and 15 Schizophrenia samples. Note that oversampling was not used on the test set in order to avoid biasing our metric of test accuracy. The confusion matrix was obtained over the test set. Furthermore, the accuracy and other indicators were calculated.

**Table 2:** Dataset Split

Category	Training	Test	Total
Healthy	20	15	35
Schizophrenia	266	15	281

The above procedure was run 10 times to avoid bias driven by random factors. The dataset split was reset for each run. Finally, the results of each test set were summarized, and the mean and standard deviation of all 10 runs were reported.

### PANSS prediction

Transfer Learning (TL) cannot be used in PANSS prediction because the pretrained network is designed for image classification, whereas PANSS prediction is a regression problem. We attempted to apply TL in PANSS prediction, but the network did not converge. In light of this, we developed a convolutional neural network from scratch to predict PANSS positive, negative, general psychopathology, and total scores, respectively. Although the factors model of PANSS are widely acknowledged recently [16, 17], while the subscales of PANSS are more widely used in real-world clinical assessment and RCT research. Therefore, in this study, we predict the total score and subscale score of PANSS, to get a better application in the clinical assessment.

The network structure established is shown in Table 3. As the same in our TL ResNet, input images were resized to 224*224*3. Batch normalization was employed to speed up the training by alleviating the covariance shift. Table 4 shows the training parameters.

**Table 3:** Network structure of PANSS prediction model

Serial Number	Name	Output Size
1	ImageInputLayer	224*224*3
2	batchNormalizationLayer	
3	convolution2dLayer	55x55x8
4	batchNormalizationLayer()	
5	convolution2dLayer	27x27x16
6	batchNormalizationLayer();	
7	convolution2dLayer	13x13x32
8	averagePooling2dLayer	7x7x32
9	fullyConnectedLayer	1x1x1
10	regressionLayer

**Table 4:** Training parameters of PANSS prediction model

Name	Value
Optimizer	Adam
MaxEpochs	30
InitialLearnRate	0.01
MiniBatchSize	128
LearnRateSchedule	piecewise
LearnRateDropPeriod	10
LearnRateDropFactor	0.5
L2Regularization	0.005

We selected 244 subjects with valid PANSS scores out of 281 subjects. The remaining 37 subjects were excluded as they did not complete the PANSS test. Of the 244 subjects, 3/4 were used for training, and the remaining subjects were used as the test set.

## Results

### Demographic information

There were 246 schizophrenia patients (70 females) and 35 HCs (14 females) included in the study and the course of disease for schizophrenia patients was 34.54 (SD = 10.76) years. There were no significant differences in gender (χ^2^ = 1.949, *p* = 0.163), while significant differences were found in age (t = 2.790, *p* = 0.006, schizophrenia:60.98 ± 9.789; HC: 56.00 ± 10.505) and education years (χ^2^ = 8.603, *p* = 0.035) between the two groups. The PANSS score of schizophrenia patients was 55.37 (SD = 13.71), the score of Positive, Negative, and General Psychopathology scale was 11.33 ± 4.46, 16.51 ± 6.41, and 27.53 ± 6.39 respectively. 95.53% of the patients were on second-generation antipsychotics (SGA) only (Olanzapine: 30.08%; Quetiapine: 18.29%; Clozapine: 40.65%; Risperidone: 25.20%; Aripiprazole: 13.82%; others: 12.60%), 0.81% of the patients were on first-generation antipsychotics (FGA) only, 99.18% of the patients were on both SGA and FGA. 55.28% of the patients were with single-drug treatment, 44.72% of the patients were with combined pharmacotherapy (two drugs: 44.31%; three drugs: 0.41%).

### Color and stroke analyses in painting from patients

**Fig. 4:**
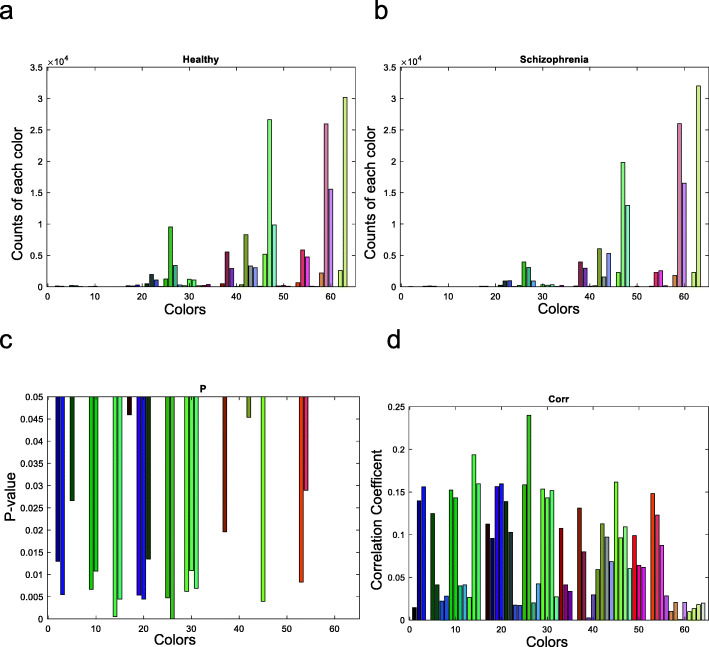
Color analysis. (**a**) shows the counts of each color used by healthy subjects; (**b**) shows the counts of each color used by schizophrenia; the X-axis represents the different colors, and the y-axis represents the counts of each color. (**c**) shows the *p-*value and here we only show those colors with *p*-values less than 0.05. (**d**) the correlation analysis of each color with schizophrenia

We have plotted the color use distribution and frequency from patients and HCs (Figure 4ab). To investigate whether the HC showed significantly different color patterns compared to patients, we applied correlation analysis with whether the subjects are schizophrenia and the color they used. The correlation coefficient and *P*-value are shown in Figs. 4(d) and (c), respectively. The patient group tends to use less color in paintings compared to HCs, an effect that was consistent across most color choices.

The “accelerator space” for paintings by the HCs and schizophrenia patients was shown in Fig. 2(b). A yellow-red pseudo color was added to reflect the values. The x-axis represented the distance between the center of the painting and the line they draw. The y-axis represented the angle of their line. The color in Fig. 2(b) represented the spatial locations of the detected line. Schizophrenia patients draw more lines around the center of the painting compared with HC. Besides, the lines of patients were more disordered relative to HC (Figs. 2cd, c shows the *p*-values, where we set the strictest threshold *p* < 0.001, due to mitigating the effect of multiple comparisons. Figure 2d illustrates the correlation coefficients, where we only show the correlation coefficients greater than 0.3).

### Schizophrenia detection results of using ResNet

**Fig. 5:**
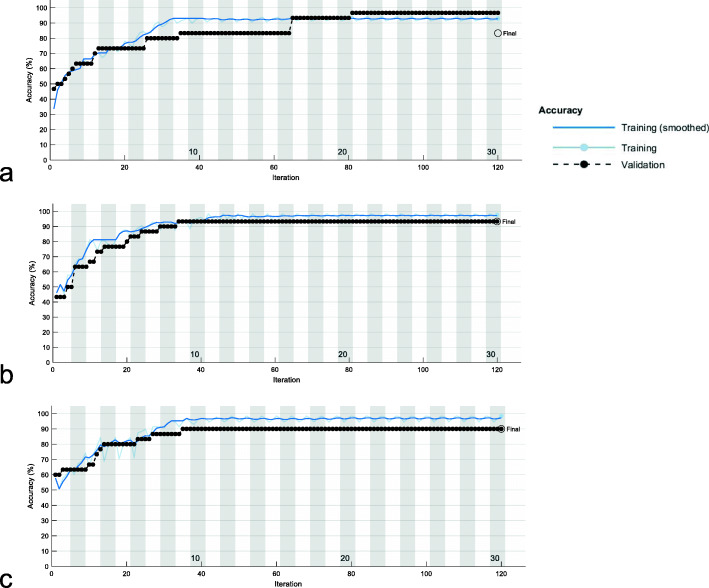
Transfer learning results via ResNet18. Training Curves with test accuracy of (**a**) 96.67%; (**b**) 93.33%, (**c**) 90.00%

The overall average test accuracy of classification for schizophrenia patients and HCs using ResNet-18 was 90.33%, which indicates high accuracy of classification. The detailed test accuracy of the 10 runs were 96.67% (2 runs out of 10 runs), 93.33% (1 run out of 10 runs), 90.00% (4 runs out of 10 runs), 86.67% (2 runs out of 10 runs), and 83.33% (1 run out of 10 runs), respectively (more details see Table 5 and Fig. 5). In this paper, validation and test are exchangeable since we used the hold-out validation technique. The training set and validation set are independent of each other. The training set was used to train the weights of the deep neural network model, and the validation set was used to calculate unbiased performance.

**Table 5:** Results of 10 runs

Number of runs	Accuracy
2	96.67
1	93.33
4	90.00
2	86.67
1	83.33
10 (in total)	90.33

### PANSS prediction

**Fig. 6:**
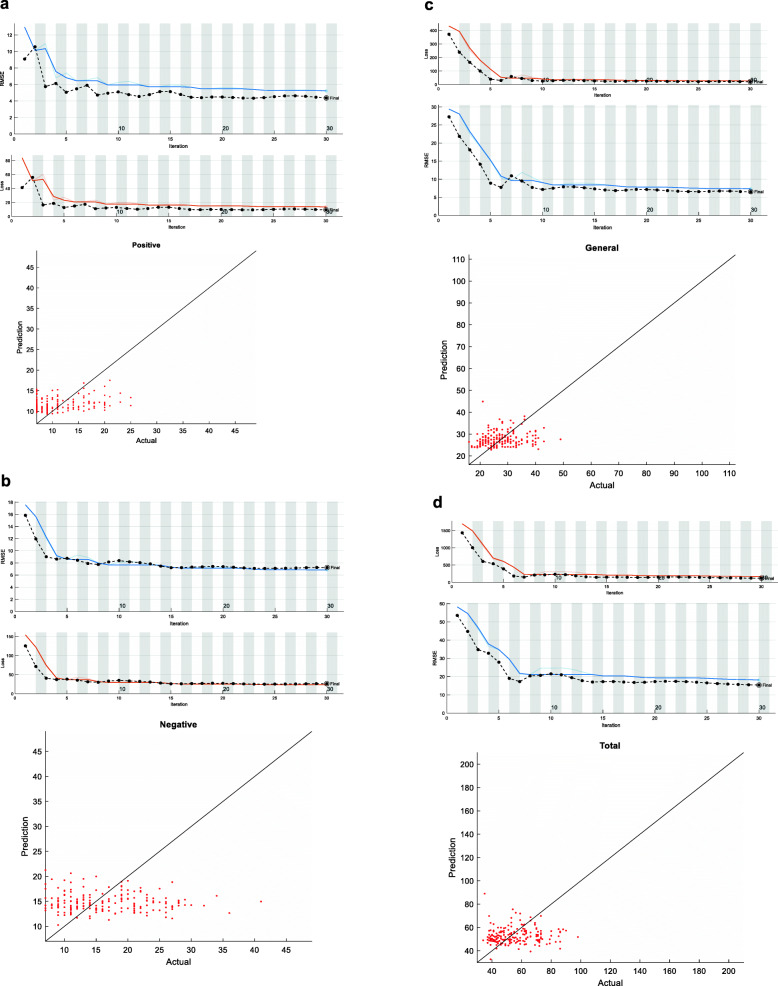
Deep learning to predict PANSS score (**a**) PANSS positive subscore, the test RMSE error is 4.57; (**b**) PANSS negative subscore, the test RMSE error is 6.78; (**c**) PANSS general psychopathology subscore, the test RMSE error is 7.38; (**d**) PANSS total score, the test RMSE error is 14.97. For the right part images, the x-axis means the actual PANSS score and the y-axis stands for the predicted PANSS score

The deep-learning-based prediction for the PANSS score shows high accuracy, The Root Mean Square Error (RMSE) of the prediction is 15.39 for the PANSS total score, 4.36 for the Positive subscale, 7.24 for the Negative subscale, 6.48 for the General Psychopathology subscale. The lower the RMSE, the more accurate that prediction is. The model-training convergence curves and the comparison of actual result and prediction results on the test set, of the positive, negative, general psychopathology, and PANSS total scores were shown in Fig. 6a-d, respectively. The closer the point approximates to the diagonal line, the more correct that prediction is.

## Discussion

Color painting courses are routinely adopted in the clinical rehabilitation program for chronic psychotic patients. Here we reported that the potential to discriminate clinical schizophrenia patients from HCs based on color paintings. The deep learning paradigm based on our color painting dataset allows sensitive prediction of clinical symptom severity for chronic schizophrenia patients. This approach, therefore, provided novel options in proper evaluation of the rehabilitation stage for patients and has the potential to be adopted in home environment, especially for circumstances that systemic clinical interview is not available.

Schizophrenia is known to be associated with altered perception [1]. Studies have examined the neural mechanisms underlying these changes, which include aberrant neural transmission (especially dopamine), altered information gating ability, and cortical dysfunctions. Indeed, the decreased use of certain colors by schizophrenia patients could reflect either the shifted perception ability or the associated mood states. Besides, in this study, the features of color painting can predict the total score and subscale score of PANSS well, suggesting that impaired visual perception is closely related to their symptom severity [6]. The current results are robust, and training the paradigm using additional paintings is expected to increase the sensitivity and specificity for clinical diagnosis.

Additionally, the stroke changes seen in the clinical group could partly be attributed to the psychomotor changes in schizophrenia patients [18]. The schizophrenia patients showed more disordered and shorter lines compared to HC, which suggested the impaired fine motor ability [19]. The impaired fine motor ability has been described as a risk factor for schizophrenia [20]. Therefore, the current classification model could valuably contribute to detecting beginning schizophrenia. Fine motor ability is also affected by chronic intake of antipsychotics, which might impact the nigral dopaminergic system [21]. It is still possible that the changes in stroke would be improved gradually with prolonged medication, or with targeted treatments that improve the psychomotor functions in these patients. It will also be helpful to include more patients at acute phases of treatment and to test if our paradigm suits well for more severe and acute patients of schizophrenia.

The study is limited by the number of patients and the limited period of longitudinal data collected. Besides, Since the patient population is a long-term hospitalized patient, long-term use of antipsychotic medications may have an impact on their color perception or fine motor ability, so more first-episode schizophrenia patients should be included to verify the model in the future. It will also be necessary to expand the color painting design, together with more choices of painting options that eliminate potential individual differences due to education or cultural background. Last but not least, it will be interesting to test the cross-disease specificity of this paradigm by recruiting depression patients and bipolar mania patients for comparison, for instance.

## Conclusion

In conclusion, the study firstly reported the potential for computational analyses of color painting from clinical psychotic patients. These findings have potential in the development of novel clinical evaluation tools based on artificial intelligence.

## Data Availability

The datasets used and/or analysed during the current study are available from the corresponding author on reasonable request.
